# A multiplex nested PCR for the detection and identification of *Candida* species in blood samples of critically ill paediatric patients

**DOI:** 10.1186/1471-2334-14-406

**Published:** 2014-07-21

**Authors:** Cleison Ledesma Taira, Thelma Suely Okay, Artur Figueiredo Delgado, Maria Esther Jurfest Rivero Ceccon, Margarete Teresa Gottardo de Almeida, Gilda Maria Barbaro Del Negro

**Affiliations:** 1 Laboratory of Medical Mycology (LIM-53), Clinical Dermartology Division, Hospital das Clínicas da Faculdade de Medicina da Universidade de São Paulo (HCFMUSP) and Instituto de Medicina Tropical da Universidade de São Paulo (IMT-USP), Av. Dr. Enéas Carvalho de Aguiar, 500, Andar térreo, Predio 2, CEP, 05403-900 São Paulo, SP, Brazil; 2 Laboratory of Seroepidemiology and Immunobiology, IMT-USP, São Paulo, SP, Brazil; 3 Pediatric and Neonatal Intensive Care Units, Instituto da Criança, HCFMUSP, São Paulo, SP, Brazil; 4 Microbiology Laboratory, Department of Dermatologic, Infectious and Parasitic Diseases, Faculdade de Medicina de São José do Rio Preto (FAMERP), São José do Rio Preto, SP, Brazil

**Keywords:** Candidaemia, *Candida* spp, Multiplex PCR, ICU, Paediatric, Diagnosis

## Abstract

**Background:**

Nosocomial candidaemia is associated with high mortality rates in critically ill paediatric patients; thus, the early detection and identification of the infectious agent is crucial for successful medical intervention. The PCR-based techniques have significantly increased the detection of *Candida* species in bloodstream infections. In this study, a multiplex nested PCR approach was developed for candidaemia detection in neonatal and paediatric intensive care patients.

**Methods:**

DNA samples from the blood of 54 neonates and children hospitalised in intensive care units with suspected candidaemia were evaluated by multiplex nested PCR with specific primers designed to identify seven *Candida* species, and the results were compared with those obtained from blood cultures.

**Results:**

The multiplex nested PCR had a detection limit of four *Candida* genomes/mL of blood for all *Candida* species. Blood cultures were positive in 14.8% of patients, whereas the multiplex nested PCR was positive in 24.0% of patients, including all culture-positive patients. The results obtained with the molecular technique were available within 24 hours, and the assay was able to identify *Candida* species with 100% of concordance with blood cultures. Additionally, the multiplex nested PCR detected dual candidaemia in three patients.

**Conclusions:**

Our proposed PCR method may represent an effective tool for the detection and identification of *Candida* species in the context of candidaemia diagnosis in children, showing highly sensitive detection and the ability to identify the major species involved in this infection.

## Background

*Candida* is the main etiological agent of nosocomial opportunistic mycoses worldwide and is associated with high mortality rates, especially in patients with underlying diseases [1,2]. *Candida* species are the third most common pathogen isolated from bloodstream infections of preterm infants and the fourth most common pathogen in paediatric intensive care unit (ICU) patients [3-5]. The incidence of invasive candidiasis among critically ill paediatric patients has increased during the last two decades, and the outcome of *Candida* infections is particularly poor in very low birth weight infants due to central nervous system involvement, leading to neurodevelopmental impairment [6].

*Candida albicans* is the most frequent cause of candidaemia in paediatric patients, followed by *C. parapsilosis* in neonates. *Candida tropicalis* has been frequently reported in bloodstream infections of neonates and children in Latin America and some regions of southern Asia [7,8].

Some groups of paediatric patients are especially predisposed to candidaemia, including preterm infants, children with haematological malignancies, stem cell and solid organ transplant recipients, children requiring a prolonged hospital stay in the ICU, and children undergoing medium or large surgeries. There are also risk factors associated with candidaemia, such as the use of central venous or arterial catheters, broad-spectrum antibiotics, and parenteral nutrition [3,5].

The gold standard of laboratory diagnosis remains the isolation of *Candida* species by blood culture. However, the sensitivity of blood cultures is approximately 50%, and the collection of small blood volumes in neonates and young children further decreases this sensitivity [9]. In addition, a time period of 48 to 96 hours is required for the identification of *Candida* species by blood culture [10]; thus, faster detection of *Candida* spp. in the blood would expedite the initiation of treatment, improving the prognosis of patients [11]. In an attempt to shorten the time required for detection of candidaemia, several groups have developed non-culture methods based on the polymerase chain reaction (PCR), which has proved to be highly sensitive and specific for the detection of *Candida* DNA in the blood samples of at-risk patients [12,13]. In addition, multiplex PCR designed to detect different targets simultaneously is time-saving and more cost-effective than the standard PCR [10,14].

This study aimed to develop a multiplex nested PCR method to detect and identify seven *Candida* species in peripheral blood samples of critically ill paediatric patients presenting with predisposing conditions/risk factors for the development of candidaemia. The results of PCR were compared with those of blood cultures.

## Methods

### Patients and blood samples

This research was approved by the human research ethics committee of our institutions [*Comissão de Ética para Análise de Projetos de Pesquisa do Hospital das Clínicas da FMUSP, São Paulo, SP,* Brazil and *Comitê de Ética em Pesquisa da FAMERP, São José do Rio Preto, SP*, Brazil].

This prospective study enrolled 54 consecutive paediatric patients, among whom there were 24 neonates, admitted to the ICU of two paediatric referral hospitals in São Paulo State, Brazil. The patients presented with systemic inflammatory response syndrome (SIRS) according to the International Pediatric Sepsis Consensus [15] and at least two predisposing conditions/risk factors for the development of candidaemia.

The control group included 28 children from the Pediatric Surgery Division of the *Instituto da Criança* – HCFMUSP that had no evidence of any type of bloodstream infection and had undergone minor surgical procedures during the same time. The number of studied cases and controls was determined based on the 18-month period set for the completion of the research (convenience sample).

Although all the patients had central catheters, blood samples for use in cultures and multiplex nested PCR were collected from a peripheral vein after written consent was obtained from parents or legal guardians.

### Blood cultures and phenotypic identification of *Candida* isolates

Cultures were obtained aseptically by inoculating 1-2 mL of blood samples into Bactec Peds Plus/F bottles (Becton Dickinson, Franklin Lakes, NJ, USA), which were incubated in a Bactec™ 9240 analyser (Becton Dickinson). The organisms obtained from positive cultures were identified using the Vitek ®2 system (BioMérieux, Marcy-l’Etoile, France). Blood cultures were not performed for members of the control group due to the absence of a clinical indication for this exam.

### DNA extractions and PCR to amplify the β-actin gene

DNA extractions were performed according to the techniques described by Löeffler *et al*. [16] with modifications. Briefly, 10 mL of blood lysis solution was added to 1-2 mL of blood sample, and the mixture was centrifuged for 15 minutes at 2190 × *g*. Nucleo lysis solution (1 mL) was added to the cell pellet, and the solutions were homogenised. Recombinant lyticase (L-2524, Sigma-Aldrich, St. Louis, MO, USA) at 250 U/mL and 2% β-mercaptoethanol (Merck, Darmstadt, Germany) were added, and the tubes were maintained at 37°C for 2 hours. The tubes were then centrifuged for 20 minutes at 15,300 × *g* and the supernatant was transferred to clean tubes. The remaining steps were performed with the QIAamp DNA mini kit (QIAGEN, Hilden, Germany) according to the manufacturer’s instructions. Then, the 54 DNA samples were amplified using a pair of human β-actin gene primers to ensure the integrity of the DNA and the absence of amplification inhibitors.

### *Candida* prototype strains

DNA was extracted from seven *Candida* prototype strains (*C. albicans* ATCC18804, *C. glabrata* ATCC2001, *C. parapsilosis* ATCC22019, *C. tropicalis* ATCC 200956, *C. krusei* ATCC6258, *C. lusitaniae* ATCC66035, and *C. pelliculosa* ATCC8168) using a previously described method [17], and these samples were used as positive controls.

### PCR primers

The fungus-specific universal oligonucleotides ITS1 and ITS4 [18] were used as outer primers. In the second amplification, the previously described inner primers for *C. albicans*, *C. glabrata*, *C. parapsilosis* complex, *C. tropicalis*, and *C. krusei* were used [18,19]. The primers for *C. lusitaniae* and *C. pelliculosa* were designed with the PrimerQuestSM and Primer-BLAST tools. The primer sequences are listed in Table 1.

**Table 1:** Primers employed in the nested multiplex PCR amplifications

**Primers**	**Sequences (5’ → 3’)**	**Amplicons**	**References**
ITS 1/4	F- TCCGTAGGTGAACCTGCGG	Variable	[18]
	R- TCCTCCGCTTATTGATATGC		
CALB 1/2	F- TTTATCAACTTGTCACACCAGA	272 bp	[18]
	R- ATCCCGCCTTACCACTACCG		
CGL 1/2	F- TTATCACACGACTCGACACT	423 bp	[18]
	R- CCCACATACTGATATGGCCTACAA		
CTR 1/2	F- CAATCCTACCGCCAGAGGTTAT	357 bp	[18]
	R- TGGCCACTAGCAAAATAAGCGT		
CPAR 3/2	F- GCCAGAGATTAAACTCAACCAA	297 bp	[18]
	R- CCTATCCATTAGTTTATACTCCGC		
CKR 2/3	F- ACTACACTGCGTGAGCGGAA	362 bp	[19]
	R- ACTACACTGCGTGAGCGGAA		
CLU 1/2	F- GCGATACGTAGTATGACTTGCAG	137 bp	*
	R- GATATTTCGGAGCAACGCCTAACC		
CPEL 1/2	F-GAACTTTGCTTTGGGTGGTGAG	160 bp	*
	R- CTTCATCGTTGCGAGAACCAAG		

F = forward; R = reverse; CALB = *C. albicans*; CGL = *C. glabrata*; CTR = *C. tropicalis*; CPAR = *C. parapsilosis* complex; CKR = *C. krusei;* CLU = *C. lusitaniae;* CPEL = *C. pelliculosa.*

* = primers designed in the current study; bp = base pairs.

### Multiplex nested PCR and detection of amplification products

To avoid carryover contamination of the assays, reaction mixes, DNA extractions and amplifications were set up in separate rooms equipped with safety cabinets [20].

The first round of amplification was carried out in a 25 μL reaction mixture containing 1× PCR buffer (Invitrogen, Carlsbad, CA, USA), 200 μM dNTP (GE Healthcare, Buckinghamshire, UK), 1.5 mM MgCl_2_, 0.2 μM each primer (ITS1 and ITS4), 1.25 U Platinum *Taq* DNA polymerase (Invitrogen), and 50 ng of genomic DNA from blood samples, which served as the DNA template. The PCR cycling conditions were as follows: an initial denaturation step of 5 min at 95°C followed by 35 cycles of 45 s at 95°C, 45 s at 50°C, and 45 s at 72°C, with a final extension of 5 min at 72°C. The reactions were carried out in a Veriti thermocycler (Applied Biosystems, Carlsbad, CA, USA).

The second round of amplifications were performed in two separate assays: assay 1, containing primers CLU, CTR, CALB, and CGL at concentrations of 0.1 μM, 0.12 μM, 0.2 μM, and 0.3 μM, respectively; and assay 2, containing primers CKR, CPAR, and CPEL at concentrations of 0.2 μM, 0.15 μM, and 0.12 μM, respectively. In both assays, 2 μL of a 1:100 dilution of the ITS PCR product was used as the DNA template. This template was mixed with the inner primers and 5% dimethyl sulfoxide (Merck, Darmstadt, Germany) to fresh reaction mixtures in a total volume of 25 μL. The amplifications were carried out in the same thermocycler under the following conditions: an initial denaturation step of 5 min at 95°C, 10 cycles of 45 s at 95°C, 45 s at 67-58°C (touchdown), and 45 s at 72°C followed by 20 cycles of 45 s at 95°C, 45 s at 58°C, and 45 s at 72°C, with a final extension of 5 min at 72°C.

In all experiments, negative controls containing sterile water instead of genomic DNA and positive controls containing *Candida* DNA were tested.

The nested PCR products were detected on 2.5% agarose gels stained with GelRedâ„¢ (Biotium, Hayward, CA, USA) and visualised under a UV transilluminator apparatus (UVITEC, Cambridge, UK).

### Multiplex nested PCR detection limit and specificity

To determine the detection limit for *Candida* DNA in clinical specimens, nested amplifications were performed with genomic DNA extracted from 1 mL of a blood sample obtained from a healthy subject spiked with the seven *Candida* species DNA ranging from 1.5 ng to 15 fg (one *Candida* genome corresponds to approximately 37 fg of DNA). Each of the *Candida* species was tested separately [21].

The PCR specificity was evaluated by testing DNA samples from microorganisms that are common etiologic agents of neonatal and paediatric sepsis, such as *Staphylococcus aureus*, *S. epidermidis*, *Streptococcus pyogenes*, *Escherichia coli*, *Enterococcus faecalis*, and *Pseudomonas aeruginosa*. The DNA samples of other *Candida* species such as *C. guilliermondii*, *C. kefyr*, *C. famata*, *C. dubliniensis* and *C. haemulonii*, as well as some non-*Candida* fungi (*Cryptococcus* sp., *Trichosporon* sp., *Rhodotorula* sp., *Aspergillus* sp., and *Fusarium* sp.) were also tested.

### Sequencing of the PCR products

To certify that the amplified products corresponded to *Candida* sequences, the PCR products generated from the blood samples that yielded negative blood cultures were sequenced on an ABI 3730 DNA apparatus (Applied Biosystems). Sequence alignments were performed with ClustalW2, and the nucleotide sequence consensus was compared with those of other *Candida* strains available in GenBank using the BLAST tool.

### Statistical analysis

Statistical analysis of the results was carried out with SPSS software (13.0). The McNemar test was employed.

## Results

The detection limit of the multiplex nested PCR for the seven tested *Candida* species was 150 fg, or the equivalent of four *Candida* genomes/mL of blood. Regarding the specificity, the bacteria, other *Candida* species and non-*Candida* fungi did not produce any amplification products.

Among the 54 enrolled patients, 32 were males and 22 were females, with ages ranging from six days to 16 years. All patients had central venous catheters and had received broad-spectrum antibiotics for more than 96 hours at the time of blood sampling. Subsequently to the blood collections, 38 patients (70.4%) initiated presumptive antifungal therapy due to the suspicion of fungal infection.

**Figure 1:**
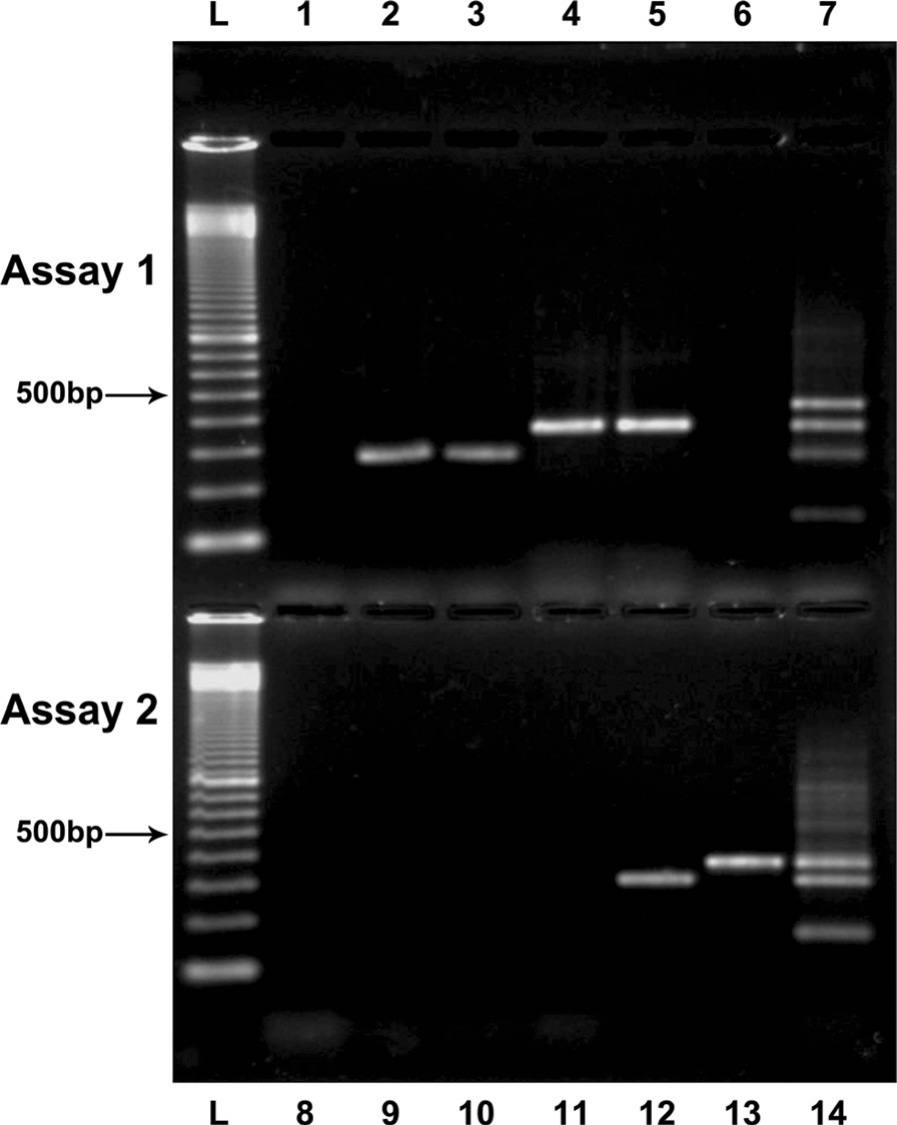
**Agarose gel showing the nested multiplex PCR products obtained from patients after amplification with primers for assays 1 and 2.** Lane L – 100 bp molecular weight marker (GE Healthcare); Lanes 1 and 8 – Negative controls; Lanes 2 and 9 – Patient 2 (*C. albicans*); Lanes 3 and 10 – Patient 8 (*C. albicans*); Lanes 4 and 11 – Patient 11 (*C. tropicalis*); Lanes 5 and 12 – Patient 13 (*C. tropicalis* and *C. parapsilosis*); Lanes 6 and 13 – Patient 7 (*C. krusei*); Lane 7 – Positive controls for assay 1: DNA from *C. glabrata* (423 bp)*, C. tropicalis* (357 bp)*, C. albicans* (272 bp) and *C. lusitaniae* (137 bp) reference strains; Lane 14 – Positive controls for assay 2: DNA from *C. krusei* (362 bp), *C. parapsilosis* (297 bp) and *C. pelliculosa* (160 bp) reference strains.

Blood from eight patients yielded positive cultures (14.8%), while *Candida* DNA was detected in 13 patients (24.0%) by the molecular assay, including the eight patients with positive cultures (Figure 1). Demographic data, clinical conditions, and the outcomes of these 13 patients are described in Table 2. The McNemar test did not uncover a disagreement between the blood cultures and the multiplex nested PCR results (p = 0.063).

**Table 2:** Demographic, clinical, and laboratory data of the 13 patients with positive PCR

**Patients**	**G**	**Age**	**Underlying disease**	**BSAT**	**CVC**	**AFT**	**Outcome**	**Blood cultures**	**Nested PCR multiplex**
1	M	19 d	Ichthyosis, prematurity	Yes	Yes	Yes	Died	*C.parapsilosis*	*C. parapsilosis*
2	M	14 d	Prematurity	Yes	Yes	Yes	Survived	*C. albicans*	*C. albicans*
3	F	44 d	Esophageal atresia	Yes	Yes	Yes	Survived	*C. albicans*	*C. albicans*
4	F	20 m	Acute lymphoblastic leukemia	Yes	Yes	Yes	Died	*C. tropicalis*	*C. tropicalis*
5	M	20 m	Non-Hodgkin lymphoma	Yes	Yes	Yes	Survived	*C. albicans*	*C. albicans*
6	F	11 m	Hydrocephalus	Yes	Yes	Yes	Survived	*C. albicans*	*C. albicans*
7	M	5 y	Disseminated medullobastoma	Yes	Yes	Yes	Died	*C. krusei*	*C. krusei*
8	F	16 m	Hydrocephalus	Yes	Yes	Yes	Died	*C. albicans*	*C. albicans*
9	M	3 y	Bone marrow transplantation, neuroblastoma	Yes	Yes	Yes	Died	Negative	*C. tropicalis* and *C. parapsilosis*^a^
10	F	15 y	Osteosarcoma, septic shock	Yes	Yes	Yes	Died	Negative	*C. parapsilosis*^a^
11	M	35 d	Congenital diaphragmatic hernia	Yes	Yes	Yes	Survived	Negative	*C. tropicalis*^a^
12	M	9 d	Congenital diaphragmatic hernia	Yes	Yes	Yes	Survived	Negative	*C. tropicalis* and *C. parapsilosis*^a^
13	M	55 d	Necrotizing enterocholitis	Yes	Yes	Yes	Died	Negative	*C. tropicalis* and *C. parapsilosis*^a^

G = gender; M = male; F = female; d = days; m = months; y = years; BSAT = broad spectrum antibiotic therapy; CVC = central venous catheter;

AFT = presumptive antifungal therapy after blood sample collections.

^a^ = Sequencing analysis showed 99-100% identity with *C. parapsilosis stricto sensu* [GenBank: JN997459.1] and 99-100% identity with *C. tropicalis* [GenBank: EU266571.1].

Regarding *Candida* species identification, PCR was 100% concordant with blood cultures. There were five patients in whom the multiplex-nested PCR was positive and the blood cultures were negative. The amplification products were submitted to sequencing analysis and showed 99 to 100% sequence identity with the *Candida* reference strains (Table 2). Among these five patients, PCR was able to identify dual candidaemia (*C. parapsilosis sensu stricto* and *C. tropicalis*) in the blood samples of three patients with negative cultures (Table 2).

The multiplex nested PCR was consistently negative when DNA samples obtained from the control group patients were amplified. In addition, the technique allowed the release of results in 24 hours, as opposed to the mean time of 72 hours for blood cultures.

## Discussion and conclusions

This study describes the development of a multiplex nested PCR method to detect and identify the seven of the most *Candida* species causing invasive candidiasis in the paediatric ICU setting: *C. albicans*, *C. parapsilosis*, *C. tropicalis*, *C. glabrata*, C*. krusei C. lusitaniae* and *C. pelliculosa*[22,23]. The primers designed to amplify these *Candida* species showed consistent and specific results (Figure 1).

Although C. *lusitaniae* is less frequently isolated from paediatric bloodstream infections, the clinical relevance of the identification of this species relates to its association with resistance to amphotericin B and worse clinical outcome [24]. Outbreaks of *C. pelliculosa* in paediatric intensive care units have already been reported in Brazil [25], reinforcing the importance of the inclusion of this *Candida* species in molecular investigations.

This multiplex nested PCR method reached the detection limit of four *Candida* genomes/mL of blood for the seven investigated species, which is an excellent analytical sensitivity considering the recommendation for candidaemia diagnostic molecular tests (i.e., detection of at least 10 CFU/mL of blood) [2,26]. Tirodker *et al*. also compared PCR and blood cultures for *Candida* detection in patients in the paediatric ICU with suspected fungemia, revealing that the molecular technique had a higher positivity. However, the single-round PCR utilised by these authors could not discriminate *Candida* species and had a detection limit of 100 CFU/0.5 mL of blood [27]. Thus, the multiplex nested PCR developed in the current study was more sensitive. Cross-reactions with other organisms aside from *Candida* as well as non-specific amplifications in the control group were not observed in the present study, indicating that this multiplex nested PCR method is both specific and sensitive.

Our molecular assay yielded more positive results than blood cultures (24.0% vs. 14.8%, respectively). Sequencing of the amplification products obtained from the five PCR-positive patients with negative blood cultures confirmed that the amplification products were from the expected *Candida* species, demonstrating the specificity of the test.

The statistical comparison between the two laboratory techniques (blood culture versus multiplex nested PCR) did not reveal a significant difference (p = 0.063), most likely due to the restricted number of patients and samples (n = 54). Nevertheless, several studies have previously demonstrated that molecular techniques perform better than culture methods [2,10,12].

In our study, *C. albicans* was the most frequently isolated species in blood cultures (five out of eight positive results), while the multiplex nested PCR identified *C. albicans*, *C. parapsilosis* complex, and *C. tropicalis* at similar frequencies (Table 2). Thus, the molecular tool allowed the identification of a range of different *Candida* species that cause bloodstream infections in these critically ill paediatric patients.

Dual candidaemia was detected in three patients only by the multiplex nested PCR method (*C. parapsilosis sensu stricto* and *C. tropicalis*). In fact, dual candidaemia has been increasingly reported with the advent of diagnostic molecular tools and may be clinically relevant in cases in which one of the detected *Candida* species is resistant to drugs usually employed in the antifungal therapy [11,12,28,29]. However, as reported in other studies [30,31], it was not possible to differentiate the dual candidaemia patients from those with only one *Candida* species with respect to the risk factors and the infection outcome, probably because of the restricted number of cases. Although *C. albicans* is often involved in mixed candidaemia episodes [29-31], this species was not present in these three particular cases. This may be related to the fact that in Brazil *C. parapsilosis* and *C. tropicalis* are the non-*C. albicans* species more frequently isolated from candidaemia episodes, particularly in paediatric ICU patients [32,33].

While detection of *Candida* DNA by the PCR assay in blood samples from patients with negative cultures can be related to transient episodes rather than true candidaemia, the finding of a positive PCR in a critically ill patient should be strongly considered due to the high potential of this eventually transient episode to rapidly turn into a systemic infection. On the other hand, the fact that our multiplex PCR assay was negative in all 28 control children, strongly suggests that this assay can be useful, in association with appropriate clinical evaluation, to rule out the presence of candidaemia and interrupt unnecessary antifungal therapy [34]. Studies with higher number of patients are necessary to determine precisely its negative predictive value.

Our multiplex nested PCR method showed other important advantages. The time to PCR results was 24 hours, while the mean time to release blood culture results was 72 hours, ranging from 48 to 96 hours considering the patients in this study. The ability to detect infection early and discriminate *Candida* species is extremely important for planning the introduction of antifungal therapy, thus improving the outcome of infected patients and reducing the hospitalisation costs [11]. In addition this assay allows for the detection of *Candida* in small volumes of blood, which is an advantage for ICU neonates and younger children [6]. However, this study was designed such that equal volumes of blood were used to perform cultures and PCR (1-2 mL) to ensure that the results were comparable.

An intrinsic limitation of studies designed to validate molecular diagnostic tools for *Candida* infections is that the gold standard method—blood culture—is a technique that lacks sensitivity [2]. Other tests that could eventually be used to confirm the molecular results, such as the β-1,3 glucan and mannan tests, also lack sensitivity and specificity [35,36].

Novel technologies have recently been exploited for rapid detection and identification of *Candida* species in bloodstream infections. Some assays based on real- time platforms have also been developed to detect candidaemia, though some limitations of sensitivity and specificity were observed [36,37]. A recently described magnetic resonance-based technology (T2Candida ®) seems to be able to detect low concentrations of *Candida* in whole blood samples (as low as one CFU/mL) in less than two hours [38]. However, further investigations are still required for validation of both methodologies in the clinical and laboratory settings.

We proposed a multiplex PCR assay targeting the ITS region of *Candida* ribosomal DNA able to identify the main *Candida* species involved in bloodstream paediatric infections with a detection limit of less than 10 CFU/mL, high specificity, and as least as sensitive as blood cultures but with a shorter turnaround time. Use of the multiplex nested PCR method also allowed the detection and identification of one or more *Candida* species in the same reaction.

## Abbreviations

PCR: Polymerase chain reaction; ICU: Intensive care unit; SIRS: Systemic inflammatory response syndrome; ATCC: American Type Culture Collection; ITS: Intergenic space region of ribosomal DNA; dNTP: Deoxynucleotide triphosphate; MgCl_2_: Magnesium chloride.

## Competing interests

The authors declare that they have no competing interests.

## Authors’ contributions

CLT contributed on the conception of the study, carried out the molecular techniques, participated in acquisition, analysis and interpretation of data, and drafted the manuscript. TSO contributed on the study design and critically revised the manuscript. AFD, MEJRC and MTGA participated in patients recruitment and acquisition of data. GMBDN coordinated the study design, contributed to the analysis and interpretation of data, and critically revised the manuscript. All authors read and approved the final version of the manuscript.

## Pre-publication history

The pre-publication history for this paper can be accessed here:

http://www.biomedcentral.com/1471-2334/14/406/prepub
